# Cyanobacterial Assemblages Inhabiting the Apatity Thermal Power Plant Fly Ash Dumps in the Russian Arctic

**DOI:** 10.3390/microorganisms13081762

**Published:** 2025-07-28

**Authors:** Denis Davydov, Anna Vilnet

**Affiliations:** Polar-Alpine Botanic Garden-Institute—Separate Subdivision of Federal Research Centre ‘Kola Science Centre’, Apatity 184209, Russia; a.vilnet@ksc.ru

**Keywords:** cyanobacteria, biodiversity, Khibiny Mountains, technogenic substrates, tailing dumps, fly ash dumps, mining production, land reclaiming

## Abstract

In the process of the work of a coal power station is formed ash and slag, which, along with process water, are deposited in the dumps. Coal ash waste dumps significantly degrade the surrounding environment due to their unprotected surfaces, which are highly susceptible to wind and water erosion. This results in the dispersion of contaminants into adjacent ecosystems. Pollutants migrate into terrestrial and aquatic systems, compromising soil quality and water resources, and posing documented risks to the environment and human health. Primary succession on the coal ash dumps of the Apatity thermal power plant (Murmansk Region, NW Russia) was initiated by cyanobacterial colonization. We studied cyanobacterial communities inhabiting three spoil sites that varied in time since decommissioning. These sites are characterized by exceptionally high concentrations of calcium and magnesium oxides—levels approximately double those found in the region’s natural soils. A total of 18 cyanobacterial taxa were identified in disposal sites. Morphological analysis of visible surface crusts revealed 16 distinct species. Furthermore, 24 cyanobacterial strains representing 11 species were successfully isolated into unialgal culture and tested with a molecular genetic approach to confirm their identification from 16S rRNA. Three species were determined with molecular evidence. Cyanobacterial colonization of coal fly ash disposal sites begins immediately after deposition. Primary communities initially exhibit low species diversity (four taxa) and do not form a continuous ground cover in the early years. However, as succession progresses—illustrated by observations from a 30-year-old deposit—spontaneous surface revegetation occurs, accompanied by a marked increase in cyanobacterial diversity, reaching 12 species.

## 1. Introduction

The reclamation of technogenic substrates is a critical concern for numerous industrial regions. In Russia, as in several countries, coal is still the most widely used source of energy for electricity generation [1]. A residue of coal combustion, fly ash is classified as solid waste that is collected in ash dumps [2]. With only 10% of coal ash being utilized in Russia [3], there is a pressing need to improve recycling efforts, given the vast amounts generated annually. These substrates have a direct impact on the environment since unprotected, vegetated surfaces are prone to severe wind and water erosion, which contaminates the soil, water, and air and exacerbates the sanitary and epidemiological conditions of nearby towns. Particles of ash can be suspended in the air for long periods.

Nowadays, managing and restoring damaged areas is a formidable task. Northern ecosystems, in particular, are characterized by slow processes of energy and mass exchange due to climatic features, which makes them especially vulnerable to anthropogenic impacts [4]. The significance of the issue regarding the reclamation of technogenic substrates is attributed to the escalating pressure on northern ecosystems resulting from the increase of industrial activity.

The predominant approach for remediating anthropogenic waste sites in the Murmansk region is cultivating plants that stabilize the substrate [5]. Substantial regions remain unaddressed by reclamation, primarily owing to the exorbitant costs of the employed technologies or the prevailing high toxicity of the substrates. The problem with waste heap reclamation is that the wastes may contain toxic elements (heavy metals), are highly alkaline, are poor in organic compounds, and retain water poorly, which makes natural restoration of vegetation difficult. Exploring cyanobacterial strains that exhibit resistance to heavy metals for the development of technologies aimed at slag overgrowing appears promising.

Phycoremediation, a technique that uses algae to remediate contaminated environments, is one of the most promising applications. A very promising biotechnological strategy that is gaining more scientific attention for its potential to reduce soil deterioration is the use of cyanobacteria as soil inoculants. In a variety of environmental conditions and on a range of soil textures, cyanobacterial strain inoculation has demonstrated the capacity to markedly enhance the physicochemical characteristics of degrading soils [6,7,8,9,10,11,12]. Inoculation with cyanobacteria increases the stability of sediments against water and wind erosive activity. The synthesis of extracellular matrix by cyanobacteria facilitates biofilm development and surface anchoring. Their growth results in the creation of self-sustaining microbial communities known as biocrusts. These communities guarantee the deposition of vital organic materials, mainly carbon and nitrogen, in substrate and start the process of primary soil formation.

It has been shown that cyanobacteria have an important impact on the development of fertility of technogenic ecotopes and stimulate further development of vegetation cover [13,14]. Cyanobacteria are among the most common and important producers in the ecosystems of the Murmansk region, especially in the formation of “pioneer” communities colonizing bare substrates [15]. They promote the growth of other plants by releasing nutrients like carotenoids, vitamins, and phytohormones into the environment, along with nitrogen, phosphorus, and potassium [16,17]. Growing quickly and being able to be cultivated in huge quantities while inoculating substrates with biomass are two crucial traits of cyanobacteria. One of the easiest and least expensive techniques in a lab setting is growing cyanobacteria biomass [18].

The aim of this work was to screen the diversity of cyanobacteria growing on technogenic substrates in the Murmansk region, and their isolation, purification, and species identification. This stage of work is necessary for further research on selecting the most productive strains in laboratory and field experiments on remediation.

## 2. Materials and Methods

### 2.1. Description of Study Sites

The slag heaps of the Apatity thermal power station were used as the object for strain’s screening. The Apatity power station is a combined heat and power plant (CHP). The plant was launched in 1959 and includes eight energy (steam) boilers and five turbo-generators. Approximately 303 thousand tonnes of coal are consumed annually [19]. As a result of burning crushed stone coal into a powdery state, ash and slag are formed, which, along with process water, are deposited in the dumps.

**Figure 1:**
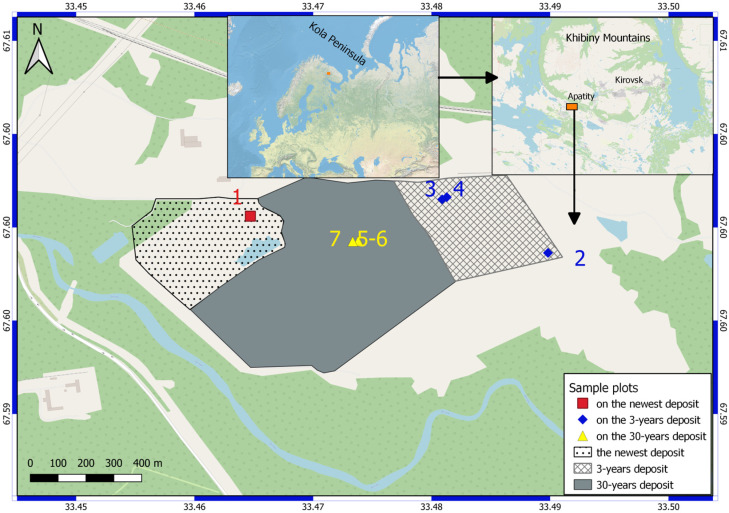
The position of the sample plots of cyanobacterial strains; numbers of sample plots as outlined in Table 1.

**Figure 2:**
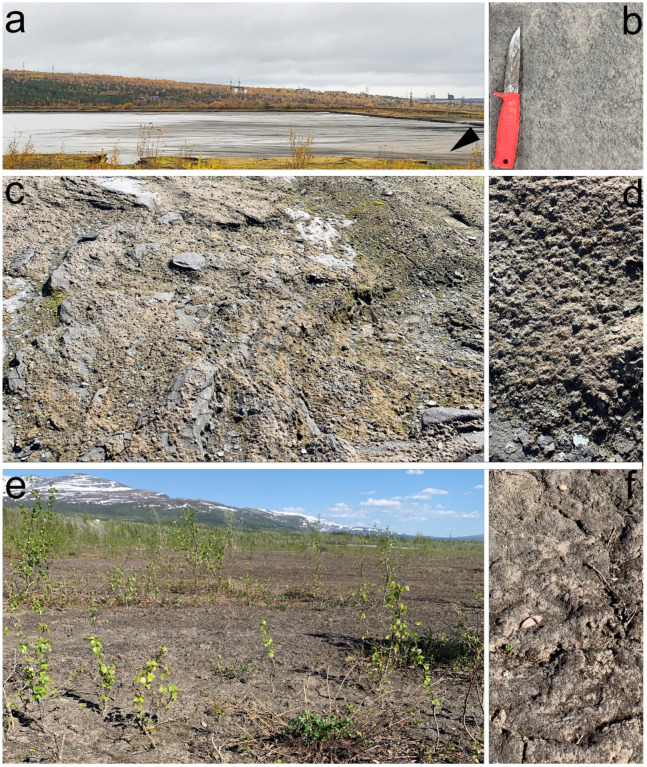
Sampling site documentation on an ash dump of Apatity CHP: (**a**) the newest dump, where pulp continues to be deposited this year; the sampling location is marked with a triangle; (**b**) thin biological soil crusts on the ground of fresh sludge; (**c**) a 3-year-old dump; (**d**) biological soil crusts with bryophytes on the 3-year-old dump; (**e**) a 30-year-old dump with active overgrowth, including woody vegetation; (**f**) thick biological soil crusts on the ground of an old dump.

The ash dumps of the Apatity CHP are piled on the plain in the foothills of the Khibiny Mountains in the valley of the Belaya River. The study area is located in the central part of the Murmansk region, in the subzone of the northern taiga. Algae collection was carried out at three localities (see Figure 1 and Figure 2, Table 1).

Sampling site No. 1 is located in a new dump, where pulp continues to be deposited this year (Figure 2a). Biological soil crusts have formed on the ground under a tube that deposits new sludge (Figure 2b).

Sampling sites No. 2–4 are located on a dump that was deposited 3 years ago (Figure 2c,d).

Sampling sites No. 5–7 are located on an old dump, which covers an area of about 47 hectares—this is a conserved dump that was filled in 1990 [20,21] (Figure 2e,f). Active overgrowth is occurring on its surface, including woody vegetation.

At the ash dumps of the thermal power plant, the composition of the substrate is determined by the minerals of the fuel used. The main mass of the waste consists of SiO_2_ (about 50%), Al_2_O_5_ (17–20%), and Fe_2_O_3_ (8–13%), CaO (2–2.4%) with significant contributions from MgO, K_2_O, Na_2_O, TiO_2_ [21,22,23,24]. In dump soils characterized by high alkaline pH (8.4), the ash has a low bulk density, high surface area, and light texture. The mineral fraction of the waste primarily consists of fine sand (0.25–0.05 mm, 29–31%) and coarse silt (0.05–0.01 mm, 56–59%), with a minor proportion of clay particles (<0.001 mm, 2.4–3.0%). The predominance of sand-sized particles leads to rapid water filtration, while the clay and silt fractions fail to provide sufficient water-holding capacity, resulting in overall poor hydrophysical performance of the ash.

Ash samples were collected from the surface layer (up to 3 cm deep), within 10 × 10 cm plots. At site No. 1, seven samples were taken; at sites No. 4, 5, and 6, six samples per site; and at sites No. 2, 3, and 7, five samples per site. Samples were collected with a knife, placed into sterile kraft paper bags, and delivered to the laboratory on the same day.

### 2.2. Culture Condition and Morphological Study

The soil suspension from each sample was inoculated onto liquid and agarized Z8 nutrient media [25]. The cultures were maintained under artificial illumination with a 16:8 light:dark photoperiod, at a temperature of 22 °C and a light intensity of 35 μmol photons m^−2^ s^−1^. Monospecies cyanobacterial cultures were obtained through sequential subculturing between solid and liquid media. After several passages, 18 unicyanobacterial strains were isolated.

Microscopic images were captured using an AxioScope A1 microscope (Zeiss AG, Oberkochen, Baden-Württemberg, Germany) with Nomarski interference contrast and an Olympus DP23 camera (Tokyo, Japan). Morphometric measurements were taken using an Olympus cells Sens Entry 3.2 (Tokyo, Japan). For morphological examination, the field samples and the three-week-old cultures were used, and each measurable characteristic of an isolate was measured 70–80 times, with various positions of the slide preparation. Initial morphological identification was performed following generally accepted keys [26,27,28].

### 2.3. DNA Extraction and Sequencing

DNA was extracted from 24 unicyanobacterial cultures using the commercial HiPure SF Plant DNA Kit (Magen, Guangzhou, China) according to the manufacturer’s protocol. The partial 16S rRNA gene and the 16S–23S internal transcribed spacer (ITS) region were amplified using a pair of primers: primer 1 (5′-CTC TGT GTG CCT AGG TAT CC-3′, [29]) and 27F (5′-AGA GTT TGA TCC TGG CTC AG-3′, [30]). PCR was carried out in 20 µL reaction volumes with MasDDTaqMIX (Dialat Ltd., Moscow, Russia) in the following amplification cycles: 3 min at 94 °C, 40 cycles (30 s 94 °C, 40 s 56 °C, 60 s 72 °C), and 2 min of final extension time at 72 °C. The amplified fragments were visualized on 1% agarose TAE gels by EthBr staining, purified using the Cleanup Mini Kit (Evrogen, Moscow, Russia), and used as a template in sequencing reactions on an Applied Biosystems 3730 DNA Analyzer (Applied Biosystems, Waltham, MA, USA) at the “Genome” Core Facility (Institute of Molecular Biology, Moscow, Russia), according to the standard protocol.

An additional internal primer (5′-GGG GGA TTT TCC GCA ATG GG-3′; [31]) was used for sequencing. For each strain, a single amplicon covering the entire 16S–23S rRNA region was sequenced without ambiguities.

The list of analyzed strains, along with voucher and GenBank accession numbers, is provided in Table 2.

### 2.4. Molecular Phylogenetic Analyses

The nucleotide sequences of 16S–23S rRNA for tested strains were assembled using BioEdit version 7.0.1 [32]. The BLAST 2.17.0 search for 16S rRNA was implemented for the 16S rRNA gene of each strain to provide preliminary taxonomic identification. Among the 24 tested strains, representatives of six different families were identified: Oculatellaceae Mai & J.R. Johansen, Nodosilineaceae Strunecký & Mareš, Wilmottiaceae Strunecký & Mareš, Microcoleaceae Strunecký, Johansen & Komárek, Nostocaceae Eichler, and Nodulariaceae Elenkin. Appropriate datasets were compiled to determine the phylogenetic affinities of the obtained strains by downloading 16S rRNA gene sequences of reference strains from GenBank, in accordance with the modern classification of cyanobacteria [33,34].

The maximum likelihood (ML) method using IQ-TREE [35] was employed to reconstruct the molecular phylogeny for each of six families based on the 16S rRNA gene, in order to determine the phylogenetic placement of the tested strains. The ML analysis was performed using the best-fit nucleotide substitution model selected by ModelFinder [36] and ultrafast bootstrapping [37] using 1000 replicates.

Nucleotide sequence similarity for the 16S rRNA gene was calculated using the formula *S* = 100 × (1 − *p*), where *p* represents the average pairwise *p*-distance. Pairwise distances were calculated in MEGA version 11 [38], using the pairwise deletion option to account for alignment gaps.

## 3. Results

A total of 18 species of cyanobacteria were identified as a result of the slag dump overgrowth (Table 3). On the basis of microscopic examination of field samples, 15 taxa of cyanobacteria were found in the area. The isolation of strains and their identification based on molecular genetic data allowed us to support identification of eleven taxa. Three taxa were not detected during direct microscopy of environmental samples but were only identified after isolation into pure culture.

**Figure 3:**
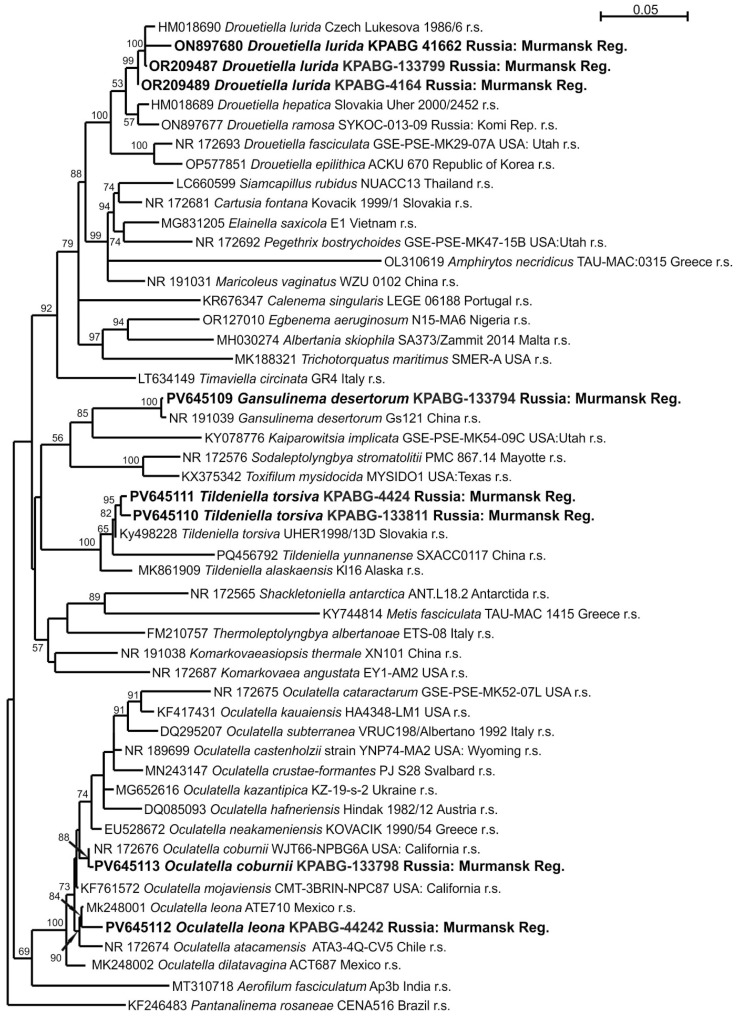
Maximum likelihood phylogram for the family Oculatellaceae based on 16S rRNA sequences. Bootstrap support values greater than 50% are shown. GenBank accession numbers and strain voucher details are provided; reference strains are marked as r.s. Samples isolated from ash dumps are shown in bold.

The eight strains analyzed using 16S rRNA sequences were identified as belonging to the family Oculatellaceae based on the BLAST algorithm. The 16S rRNA dataset for the Oculatellaceae family, comprising 51 accessions and 1324 positions, includes reference strains of the type species for 24 currently accepted genera. The genera *Drouetiella*, *Gansulinema*, *Tildeniella*, and *Oculatella* are represented by reference strains of all known species. *Pantanalinema rosaneae* from the related family Leptolyngbyaceae was selected as the outgroup taxon. The ML calculation using the GTR+F+I+G4 best fit model of nucleotide substitutions resolved in a single tree with a log likelihood of −8828.526. The resulting topology is shown in Figure 3.

**Figure 4:**
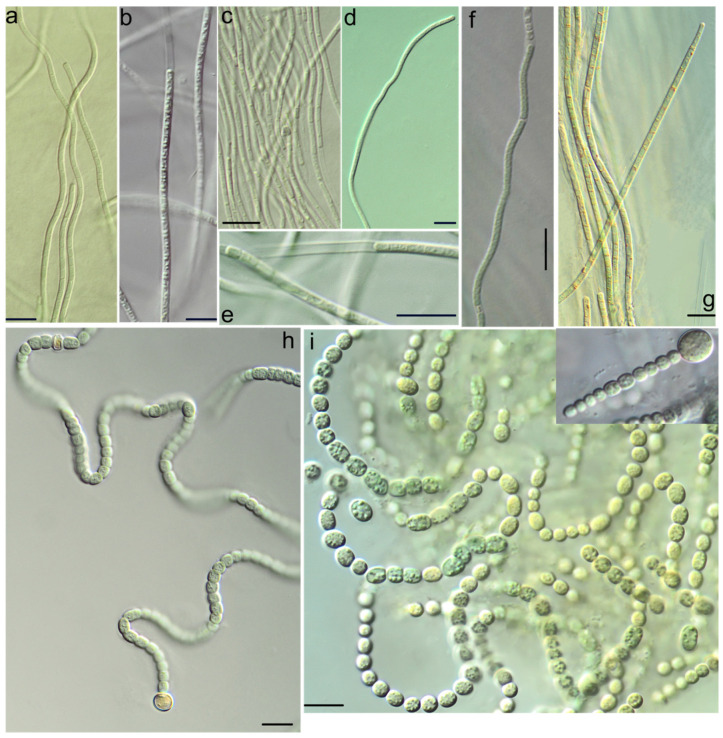
Light micrographs of cyanobacteria strains isolated from ash dumps: (**a**) *Drouetiella lurida* KPABG-4164; (**b**) *Drouetiella lurida* KPABG-41662; (**c**) *Tildeniella torsiva*-KPABG-133811; (**d**,**e**) *Oculatella coburnii* KPABG-133798; (**f**) *Nodosilinea calida* KPABG-133816; (**g**) *Anagnostidinema pseudacutissimum* KPABG-133632; (**h**) Nostocalean cyanobacterium 1 KPABG-4166; (**i**) Nostocalean cyanobacterium 2 KPABG-133801. Scale bars = 10 µm.

The strains KPABG-4164 (Figure 4a), KPABG-41662 (Figure 4b), and KPABG-133799 clustered with the reference strain of *Drouetiella lurida* Lukesová 19866. The sequence similarities among these accessions ranged from 99.00% to 99.61% (see Supplementary Materials, Table S1), not exceeding the species delimitation threshold of 98.7% [39]. Therefore, these strains are identified as *Drouetiella lurida*.

The strain KPABG-133794 formed a sister group to the reference strain of *Gansulinema desertorum* Gs121, with a 16S rRNA sequence similarity of 99.68% (Supplementary Materials, Table S2). Both its phylogenetic position and sequence similarity support its classification as *Gansulinema desertorum*.

The strains KPABG-4424 and KPABG-133811 (Figure 4c) clustered with the reference strain of *Tildeniella torsiva* UHER 1998/13D, showing 16S rRNA sequence similarities of 99.40–99.54% (Supplementary Materials, Table S3). Thus, both strains isolated from the ash dump were identified as *Tildeniella torsiva*.

The strain KPABG-133798 (Figure 4d,e) was closely related to the reference strain of *Oculatella coburnii* WJT66-NPBG6A, with a similarity of 99.83% (Supplementary Materials, Table S4). The strain KPABG-44242 clustered with the reference strain of *Oculatella leonae* ATE710, showing 99.02% similarity. Therefore, the species *Oculatella coburnii* and *O. leonae* were also identified from the dump sites.

The strain KPABG-133816 (Figure 4f) was found to be most similar to species of the genus *Nodosilinea*. The reference strains of 15 known *Nodosilinea* species, along with type species from nine other genera within the family Nodosilineaceae and the strain *Shahulinema minutum* (Oculatellaceae) used as an outgroup, were included in a 16S rRNA dataset comprising 1534 aligned positions.

**Figure 5:**
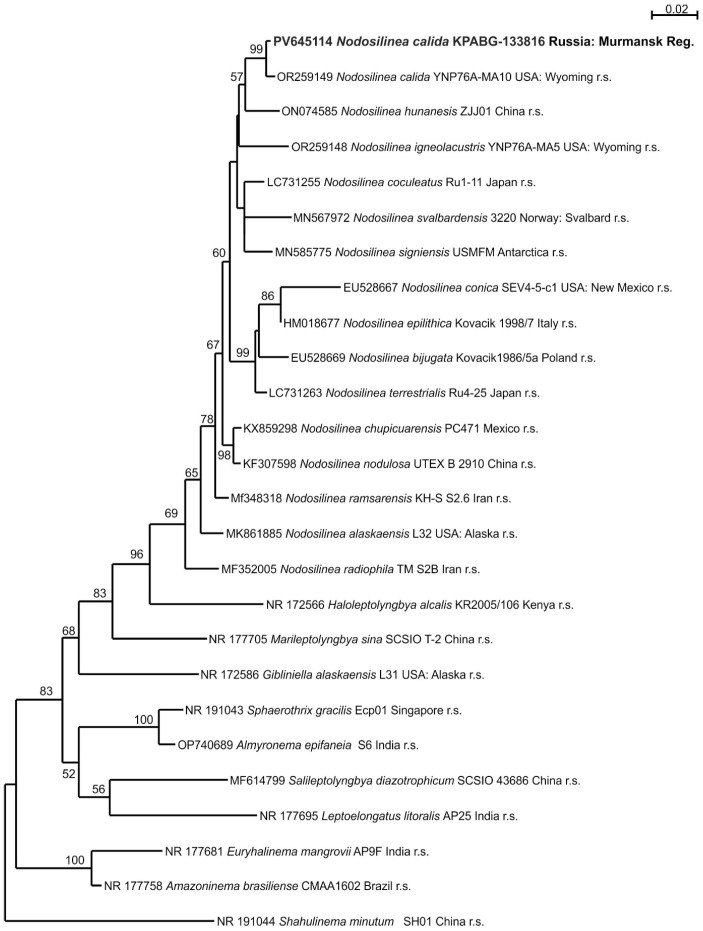
Maximum likelihood phylogram for the family Nodosilineaceae based on 16S rRNA sequences. Bootstrap support values greater than 50% are shown. GenBank accession numbers and strain voucher details are provided; reference strains are marked as r.s. Samples isolated from ash dumps are shown in bold.

A single tree with a log-likelihood of −6108.445 was reconstructed using ML analysis under the TN+F+I+G4 nucleotide substitution model, and is shown in Figure 5. The strain KPABG-133816 formed a clade with the reference strain of *Nodosilinea calida* YNP76A-MA10, with a 16S rRNA sequence similarity of 99.6% (Supplementary Materials, Table S5). Therefore, the tested strain from the dump site is identified as *Nodosilinea calida*.

Five strains (KPABG-4411, KPABG-4431, KPABG-133809, KPABG-133632 (Figure 4g), and KPABG-133633), exhibiting highly similar 16S rRNA sequences (99.7–100%) (Supplementary Materials, Table S6), were found to be closely related to several strains of the recently established genus *Anagnostidinema* [40], which currently includes twelve species, though only a few of them have available molecular data.

The 16S rRNA dataset comprises 16 accessions with 1465 aligned positions, including two epitype strains of *Anagnostidinema pseudacutissimum* (CCALA 150 and CCALA 151), *A. amphibium* RO-MK72, the reference strain of *A. visiae* LHM-M, type species from five additional genera of the family Wilmottiaceae, and *Kastovskya adunca* ATA6-11-RM4 from the closely related family Coleofasciculaceae, used as an outgroup.

**Figure 6:**
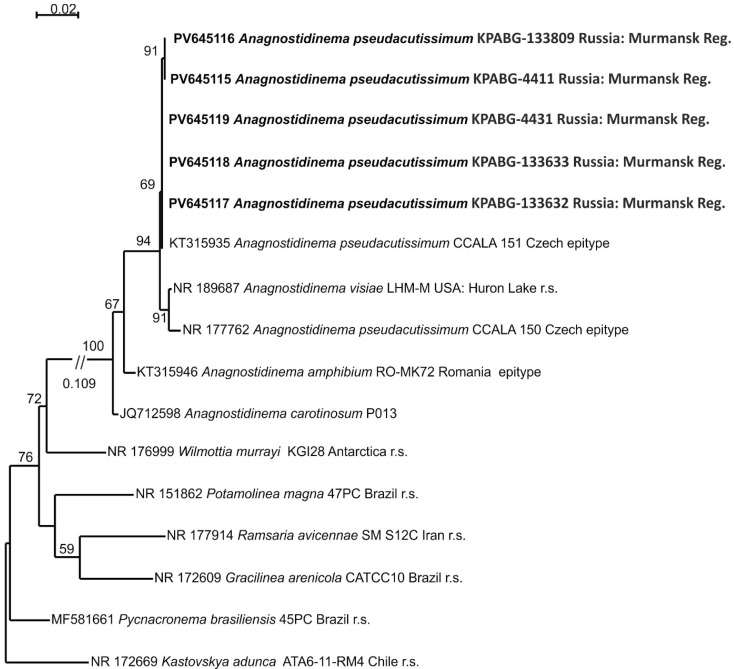
Maximum likelihood phylogram for the family Wilmottiaceae based on 16S rRNA sequences. Bootstrap support values greater than 50% are shown. GenBank accession numbers and strain voucher details are provided; reference strains are marked as r.s. Samples isolated from ash dumps are shown in bold.

The phylogenetic tree inferred through ML analysis using the TN+F+I+G4 model of nucleotide substitution, with a log-likelihood of −3990.527, is presented in Figure 6. The tested accessions formed a clade together with the epitype strains of *Anagnostidinema pseudacutissimum* and the reference strain of *A. visiae* LHM-M. These two species were differentiated by minor variations in the 16S–23S ITS rRNA region, while their 16S rRNA sequence similarity was 99.9–100% [41]. The tested strains showed 99.0–100% similarity with the reference accessions. Based on these results, the strains isolated from the dumps were identified as *Anagnostidinema pseudacutissimum*.

The strain KPABG-133810, showing over 99% similarity in a BLAST search, was affiliated with species of the genus *Microcoleus*. However, species delimitation within this globally distributed genus remains challenging and has not been robustly resolved in several dedicated studies [42,43,44]. Therefore, strain KPABG-133810 is tentatively assigned as *Microcoleus* sp.

Seven strains (236–241, KPABG-4166) were identified by BLAST as members of the family Nostocaceae, while two strains (KPABG-133801 and KPABG-133804) were assigned to the closely related family Nodulariaceae.

The family Nostocaceae had been shown to be polyphyletic based on 16S rRNA analyses [45,46]. However, a recent study by [34], based on a phylogenomic analysis of 120 housekeeping proteins across a limited sampling of genera, suggested monophyly of the families Nostocaceae, Nodulariaceae, Aphanizomenonaceae, and Tolypotrichaceae, as well as their sister-group relationships.

To avoid phylogenetic ambiguity, we produced a dataset that included key genera with type species and reference strains from all the aforementioned families. Sampling for the genus *Nostoc* followed [47]. The dataset combined 70 accessions with 1458 aligned positions, and *Inacoccus terrestris* LID-610023 (Chroococcaceae) was used as the outgroup taxon.

Phylogeny was constructed using the GTR + F+I + G4 model of nucleotide substitution, yielding a log-likelihood value of −8897.657. In Figure 5, six strains (236–241) formed a terminal clade closely related to the strain *Nostoc commune* NIES-3991. Two additional subclades of *N. commune*, comprising strain pairs SAG 1453-3 and CCAP 1453/24, as well as WY1KK1 and Ku006 NIES-3989, were recovered in more distant positions within the *Nostoc*-clade. The 16S rRNA sequence similarity among the tested strains from ash dump sites ranged from 99.26% to 100%, while their similarity to other cultured *Nostoc* strains ranged from 97.23% to 99.39% (Supplementary Materials, Table S7).

The taxonomic treatment of *N. commune* remained unresolved and required further study. However, due to the wide distribution and common morphology of this species, we provisionally identified the tested strains as *Nostoc commune* sensu stricto.

Strain KPABG-4166 was located in an unsupported relationship to the clade comprising the oligotypic genus *Dendronalium*, which included two species: *Dendronalium phyllosphericum* (strain CENA369) and *D. spirale* (strain NP-KLS-5C-PS). Both species were 99.46% 16S rRNA sequence similarity. Strain KPABG-4166 showed only 97.09–97.40% similarity to these species and could be consider a potential new species (Supplementary Materials, Table S8). We designated it as Nostocalean cyanobacterium 1 (Figure 4h).

**Figure 7:**
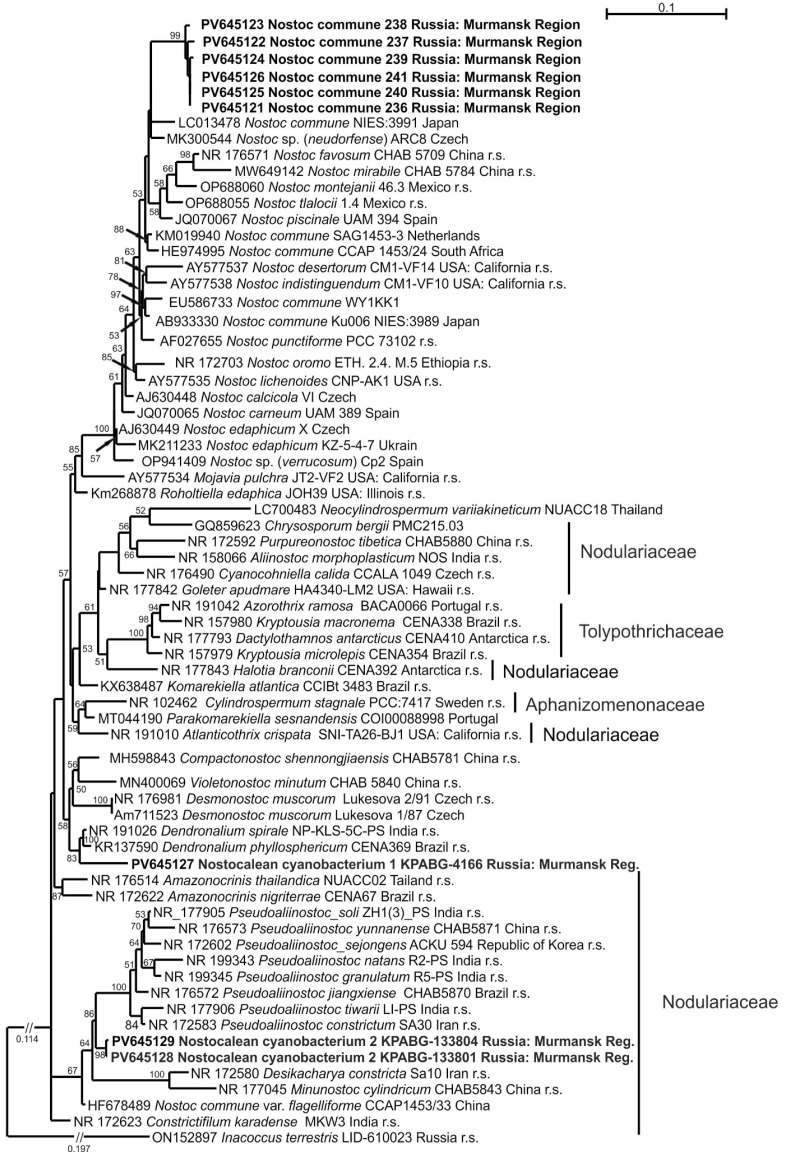
Maximum likelihood phylogram for the family Nostocaceae and related families (Nodulariaceae, Aphanizomenonaceae, and Tolypothrichaceae), as indicated on the figure, based on 16S rRNA sequences. Bootstrap support values greater than 50% are shown; the length of any cut branch is marked. GenBank accession numbers and strain voucher details are provided; reference strains are marked as r.s. Samples from slag dumps are shown in bold.

The strains KPABG-133801 and KPABG-133804 formed a clade in an unsupported position relative to the genus *Pseudoaliinostoc* of the family Nodulariaceae (Figure 7). These two strains were 99.78% similar to each other but only 95.66–97.24% similar to species within *Pseudoaliinostoc* (Supplementary Materials, Table S9). Given the observed divergence, these strains were considered to represent a separate species, here designated as Nostocalean cyanobacterium 2 (Figure 4i).

On the current-year slags, four species of cyanobacteria were identified: *Anagnostidinema pseudacutissimum*, *Microcoleus* sp., *Nodosilinea calida*, and *Nostoc* cf. pruniforme.

On the slags that had been overgrown for three years, seven species were recorded: *Aphanocapsa* sp., *Drouetiella lurida*, *Nostoc commune*, Nostocalean cyanobacterium 2, *Oculatella coburnii*, *Synechococcus elongatus*, and *Tildeniella torsiva*.

The sludge, the spontaneous overgrowth of which occurred over a period of 30 years, is characterized by a species richness of 12 taxa: *Anagnostidinema pseudacutissimum*, *Aphanothece pallida*, *A. saxicola*, *Calothrix* sp., *Drouetiella lurida*, *Gansulinema desertorum*, *Microchaete tenera*, *Microcoleus* sp., *Nostoc commune*, Nostocalean cyanobacterium 1, *Oculatella leonae*, and *Tildeniella torsiva*.

## 4. Discussion

As the study showed, the formation of cyanobacterial communities in the ash and slag dumps of the Apatity CHP begins immediately after their deposition. In the current-year slags, only three taxa were found during microscopy of fouling: *Microcoleus* sp.—morphologically corresponds to the species *M. autumnalis*; *Nodosilinea calida*; and *Nostoc* sp.—young colonies, most similar in morphology to *Nostoc pruniforme*. Analysis of grown cultures, including testing of nucleotide sequence data of 16S-ITS revealed *Anagnostidinema pseudacutissimum* (four strains), *Nodosilinea calida* (three strains), and *Microcoleus* sp. (one strain). The species *Nodosilinea calida* was isolated only at this stage of succession. As follows from the observations, substrate fixation by the first settlers does not occur during the first 1–2 years, since we did not find visible algal overgrowth on the substrate surface. The first thin crusts were detected in the three-year-old sites. In our opinion, the low species diversity can be attributed to several factors. One of them is the high drainage capacity of the substrate, leading to rapid desiccation and wind erosion of the surface layers. After natural filtration and evaporation of water from the settling tank, a finely dispersed soil layer is formed, consisting of particles less than 0.16 mm. Such an unfixed ash substrate is easily carried by the wind, leading to the formation of dust pollution in the environment. In strong winds, dust is transferred for several kilometres.

In different regions, various technologies have been tested to control wind erosion and fugitive dust emission [48,49,50,51,52,53,54,55]. Biological inoculation using cyanobacteria is widely employed due to its eco-friendliness, minimal adverse effects on soil, and high potential, as it leverages natural succession mechanisms [56,57]. However, the current literature reveals a significant knowledge gap in remediation technologies specifically adapted for Arctic and Subarctic ecosystems [58,59].

The literature demonstrates that *Microcoleus vaginatus* represents the most promising candidate for remediation of sandy and bare soil. This cyanobacterial species has been widely investigated for its applications in soil stabilization of arid regions and erosion control measures [49,60]. Several species of the genus *Microcoleus* have been recorded in various habitats across the Murmansk Region, including technogenic deposits from apatite-nepheline production [15]. These species are characterized by rapid growth rates and the ability to form dense surface biofilms. Successful cyanobacterial crust formation in low-humidity ground requires consistent surface moisture maintenance during initial colonization. Some studies suggest that establishing biological crusts with *Microcoleus vaginatus* requires maintaining soil water content at ≥20% during the crucial initial two-week period [57].

One should also consider the limitations of using individual strains for colonization, as isolated inoculation often fails to provide a stable microenvironment necessary for cyanobacteria to form functional biological crusts [56]. To mitigate the degradation risks of single-species remediation agents and enhance treatment efficacy, complex microecosystem consortia are implemented. M.A. Bowker [61] developed a biological soil crust-based restoration methodology for areas experiencing severe physical erosion processes.

Other limiting factors for the colonization of ash dumps include critical nutrient deficiencies, particularly the severe lack of nitrogen and phosphorus in fly ash, which significantly impedes natural vegetation recovery. Nitrogen is largely lost through volatilization as oxides during coal combustion, while phosphorus becomes unavailable due to high solubility and subsequent formation of insoluble compounds [62]. However, the natural atmospheric nutrient input, combined with the existing micronutrient content in slag appears adequate to support the growth of certain cyanobacterial species in this environment. In such conditions, nitrogen-fixing cyanobacterial species gain a distinct competitive advantage. These taxa play a crucial role in soil organic matter accumulation, facilitating the colonization of other organisms during later successional stages [58].

On the three-year-old dump, the species richness of cyanobacteria is higher by 1.75 times (seven species). A characteristic difference was the formation of macroscopic colonies on the upper surface layer. At this stage, such overgrowths are formed only by *Nostoc commune*. This species is found in the form of small spherical colonies up to 3–5 mm in diameter, covering individual areas of the substrate. Research has demonstrated that *Nostoc commune*-based cyanobacterial consortia can effectively remediate soils contaminated by industrial activities [63].

More overgrown areas were observed in local depressions capable of retaining moisture. In addition to cyanobacteria, an important group directly involved in fixing the technogenic substrate are organisms with a filamentous thallus organization (*Xanthonema* sp., *Heterococcus* sp., *Leptosira* sp., *Tribonema minus* (Wille) Hazen, *Xanthonema* cf. *montanum*, *Klebsormidium* cf. *pseudostichococcus*, *Geminella* cf. *minor*, *Pseudostichococcus undulatus* (Vinatzer) Van et Glaser, *Hazenia* cf. *prostrata,* and *Sarcinofilum mucosum* (Broady) Darienko et Pröschold) [64].

Two species—*Anagnostidinema pseudacutissimum* and *Microcoleus* sp.—were present in both newly deposited and 30-year-old slag, but were absent from the three-year-old successional communities. The reasons for their absence at intermediate stages of succession remain unclear and should be investigated in future studies.

Instead, the three-year communities were uniquely characterized by *Aphanocapsa* sp., Nostocalean cyanobacterium 2, *Oculatella coburnii*, and *Synechococcus elongatus*. Notably, *Drouetiella lurida*, *Nostoc commune*, and *Tildeniella torsiva* first appeared at this stage and persisted in later successional slag dumps.

Unfortunately, we were unable to follow the stages of cyanobacterial succession in more detail at this slag dump. There are no intermediate polygons between the 3-year-old and 30-year-old dumps.

The overgrowing stage observed at the 30-year-old slag dump is characterized by formed areas of closed vegetation cover alternating with unovergrown substrate. In addition to biological crusts 3 to 7 mm thick, based on *Nostoc commune*, bryophytes, lichens (*Cetraria islandica* (L.) Ach., *Stereocaulon saxatile* H. Magn., *Flavocetraria nivalis* (L.) Kärnefelt et A. Thell), clover clumps (*Trifolium repens* L.), and birch, aspen and pine undergrowth were found here.

These species—*Aphanothece pallida*, *A. saxicola*, *Calothrix* sp., *Gansulinema desertorum*, *Microchaete tenera*, Nostocalean cyanobacterium 1, and *Oculatella leonae*—were not detected during earlier successional stages. Totally, the richness of cyanobacteria in this stage increased 3 times (12 taxa) compared with the initial stage.

Overall, a fairly rich species composition of cyanobacteria was identified at the slag dump—18 taxa. The overall high species richness identified as a result of the study may be related to the high concentrations of calcium and magnesium oxides, the content of which is approximately twice as high as in the natural podzolic soils of the Murmansk region [65].

This study once again confirms the need for complex investigation that combine both the collection and identification of natural samples and the cultivation of fouling in the laboratory. Several taxa would not have been discovered if we had not conducted a direct study of fouling since these species did not grow on artificial media. On the other hand, it is also impossible to reliably identify morphologically difficult-to-distinguish species, so the use of pure cultures significantly enriched our data and allowed us to reliably determine based on 16S rRNA such taxa as *Anagnostidinema pseudacutissimum*, *Drouetiella lurida*, *Gansulinema desertorum*, *Nodosilinea calida, Oculatella* spp., *Tildeniella torsiva,* and to reveal, apparently, new species and genera of cyanobacteria for science. The study and description of the latter requires a separate study.

Among the identified species—including *Aphanothece pallida*, *A. saxicola, Microchaete tenera*, *Microcoleus* cf. *autumnalis*, *Nostoc* cf. *pruniforme*, and *Nostoc commune*—most demonstrate broad geographic distribution and considerable ecological plasticity. Of the species described in recent decades, discoveries on slag dumps seem quite non-trivial, allowing us to supplement our understanding of their habitats and ecological preferences.

*Anagnostidinema pseudacutissimum* is a species described from the freshwater lake Lunzer Obersee in Austria [66]. It is widespread in Europe: the Netherlands [67], Czech Republic, Italy [40], and Portugal [68]. It is also reported for Korea [69], India, the USA, and Antarctica [40]. In Russia, it is known only from the Far East [70]. The species appears to have a broad ecological niche, as it has been recorded in seepages, soils, and wet walls, in addition to freshwater habitats.

*Drouetiella lurida* is a worldwide-distributed species [33,71], which was identified in different ecological conditions: moist soils, stagnant water, and lake littoral zones, epilithic on a boulder in a river, and in thermal springs [28,71].

*Gansulinema desertorum* the species was known from only two desert localities in Gansu province in China [72].

*Nodosilinea calida* was described from calcareous concretions in a pool with hot water in Yellowstone National Park [73]. It has not been previously found in other habitats. As with the above taxon, this species is recorded for the territory of the Murmansk region and Russia for the first time.

*Tildeniella torsiva* was described from a limestone wall in Slovak Paradise National Park, Slovakia [74], and was subsequently reported from the Republic of Korea [75] and Ukraine (NCBI accession OR288165). These findings suggest that the species is widely distributed, at least across Eurasia. However, this is the first record of *T. torsiva* for both Russia and the Murmansk Region.

*Oculatella coburnii* was described a decade ago from granitic soils in a hot desert environment in Joshua Tree National Park, California, USA [76]. Other occurrences have been reported from Virginia Park, Utah, USA (algae culture collections at John Carroll University). *Oculatella leonae* was described from biocrusts on semi-desert soils in Atexcac, Mexico [77], and lacks additional records.

The discovery of both species on nutrient-poor artificial substrates in the Subarctic region of Russia is unexpected. However, these findings may indicate a broader distribution of these terrestrial species than previously recognized.

Based on the ecological characteristics of their habitats, *Nostoc commune*, *Drouetiella lurida*, and *Gansulinema desertorum* should be prioritized for use in phycoremediation field trials. This recommendation is supported by their demonstrated persistence across successional stages.

To effectively use cyanobacteria in reclamation projects, their cultivation productivity should be assessed, as different species require varying amounts of sunlight, organic material, and inorganic nutrients for maximum growth.

As our search for indigenous strains suitable for further use as objects for developing remediation technologies shows, the most promising species for practical use are representatives of heterocytic taxa of Nostocaceae. Such species are characterized by the following properties, which allow overcoming a number of difficulties in implementing phycoremediation: high growth rate of crops and the ability to form a large biomass, which is a necessary condition for mass cultivation of colonies for use on large areas of dumps; and the ability to fix nitrogen, which allows them to exist in conditions of a deficiency of this element in artificial soils. N_2_ fixation and exudation of organic compounds drive the successional trajectory, paving the way for plant community development. Only tolerant isolates of cyanobacteria can survive and proliferate according to their different genetic potentials for survival in the severe conditions of the fly ash substrate. Therefore, it is crucial to discover strains capable of surviving under toxic conditions. Indigenous nostocalean isolates obtained from the surveyed deposits represent promising candidates for further testing on revegetation efficacy.

Increased synthesis of exopolysaccharides, which improve the soil structure, allows binding a greater number of fine particles and increasing moisture capacity [78,79].

Another important aspect is the absence of toxin production during the formation of *Nostoc* colonies. These species degrade pollutants into simple, non-toxic, useable inorganic products, for example, carbon dioxide and water, which are further allocated in the biogeochemical cycle [80,81].

## 5. Conclusions

Cyanobacterial colonization begins immediately after ash deposition, demonstrating remarkable adaptability to extreme conditions (high Ca/Mg oxides, low N/P, fine particulate matter). Initial communities show low diversity, while mature sites develop three times richer assemblages, indicating clear successional patterns. Nitrogen-fixing nostocalean cyanobacteria, particularly *Nostoc commune*, emerge as keystone species, forming macroscopic colonies by the third year and persisting in mature communities.

The next phase of our implementation pathway involves conducting controlled stress-response assays of the top-performing cyanobacterial strains under simulated ash substrate conditions in laboratory mesocosms, followed by small-scale field trials incorporating moisture-retention amendments to evaluate their practical revegetation potential.

## Figures and Tables

**Table 1:** Location of study sites and sample plots.

Study Site	Number of Sample Plots	LongitudeE	LatitudeN	Elevation (m a.s.l.)
The newest dump	1	33.464710	67.599360	180
3-year-old dump	2	33.489820	67.598180	180
	3	33.480890	67.599900	180
	4	33.481280	67.599972	202
30-year-old dump	5	33.473850	67.598560	188
	6	33.473800	67.598520	189
	7	33.473342	67.598548	189

**Table 2:** List of cyanobacteria strains isolated from ash dumps, cultivated, and sequenced.

Taxon	Number of a Strain	Number of Sample Plots	Genbank Accession Number (16S–23S rRNA)
**Oculatellaceae**			
*Drouetiella lurida*	KPABG-133799	3	OR209487
*D. lurida*	KPABG-4164	4	OR209489
*D. lurida*	KPABG-41662	5	ON897680
*Gansulinema desertorum*	KPABG-133794	5	PV645109
*Tildeniella torsiva*	KPABG-133811	2	PV645110
*T. torsiva*	KPABG-4424	5	PV645111
*Oculatella leonae*	KPABG-44242	5	PV645112
*O. coburnii*	KPABG-133798	3	PV645113
**Nodosilineaceae**			
*Nodosilinea calida*	KPABG-133816	1	PV645114
**Wilmottiaceae**			
*Anagnostidinema pseudacutissimum*	KPABG-4411	1	PV645115
*A. pseudacutissimum*	KPABG-133809	1	PV645116
*A. pseudacutissimum*	KPABG-133632	1	PV645117
*A. pseudacutissimum*	KPABG-133633	1	PV645118
*A. pseudacutissimum*	KPABG-4431	5	PV645119
**Microcoleaceae**			
*Microcoleus* sp.	KPABG-133810	1	PV645120
**Nostocaceae**			
*Nostoc commune*	KPABG-236	7	PV645121
*N. commune*	KPABG-237	7	PV645122
*N. commune*	KPABG-238	7	PV645123
*N. commune*	KPABG-239	7	PV645124
*N. commune*	KPABG-240	7	PV645125
*N. commune*	KPABG-241	7	PV645126
Nostocalean cyanobacterium 1	KPABG-4166	5	PV645127
**Nodulariaceae**			
Nostocalean cyanobacterium 2	KPABG-133801	3	PV645128
Nostocalean cyanobacterium 2	KPABG-133804	2	PV645129

**Table 3:** List of species determined by different methods.

Species	Number of Sample Plots					
	The Newest Dump		3-Year-Old Dump		30-Year-Old Dump	
	Field Sample	Culture	Field Sample	Culture	Field Sample	Culture
*Anagnostidinema pseudacutissimum* (Geitler) Strunecký et al.		1			5	5
*Aphanocapsa* sp.			4			
*Aphanothece pallida* (Kütz.) Rabenh.					5, 6	
*A. saxicola* Näg.					7	
*Calothrix* sp.					7	
*Drouetiella lurida* (Gom.) Mai et al.			3, 4	3, 4	5	5
*Gansulinema desertorum* S. Li et F. Cai						5
*Microchaete tenera* Thur. ex Born. et Flah.					6	
*Microcoleus* sp. (cf. *autumnalis*)	1	1				5
*Nodosilinea calida* J.R. Johansen et al.	1	1				
*Nostoc* cf. *pruniforme* C. Ag. ex Born. et Flah.	1					
*Nostoc commune* Vauch. ex Born. et Flah.			4		7	7
Nostocalean cyanobacterium 1					5	5
Nostocalean cyanobacterium 2			2, 3	2, 3		
*Oculatella leonae* Becerra-Absalón et J.R. Johansen						5
*Oculatella coburnii* N. Pietrasiak et J.R. Johansen				3		
*Synechococcus elongatus* (Näg.) Näg.			4			
*Tildeniella torsiva* Mai et al.			2	2	5	5

## Data Availability

Data of specimen occurrence and culturing strains are available in the L. repository, https://isling.org/dist/#/cyano accessed on 1 June 2025.

## Supplementary Materials

The following supporting information can be downloaded at: https://www.mdpi.com/article/10.3390/microorganisms13081762/s1, Table S1: Nucleotide sequence similarity (%) for the tested strains of *Drouetiella* species from dumps (in bold) with allied accessions based on 16S rRNA gene; Table S2: Nucleotide sequence similarity (%) for the tested strains of *Gansulinema* species from dumps (in bold) with allied accessions based on 16S rRNA gene; Table S3: Nucleotide sequence similarity (%) for the tested strains of *Tildeniella* species from dumps (in bold) with allied accessions based on 16S rRNA gene; Table S4: Nucleotide sequence similarity (%) for the tested strains of *Oculatella* species from dumps (in bold) with allied accessions based on 16S rRNA gene; Table S5: Nucleotide sequence similarity (%) for the tested strains of *Nodosilinea* species from dumps (in bold) with allied accessions based on 16S rRNA gene; Table S6: Nucleotide sequence similarity (%) for the tested strains of Anagnostidinema species from dumps (in bold) with allied accessions based on 16S rRNA gene; Table S7: Nucleotide sequence similarity (%) for the tested strains of *Nostoc* species from dumps (in bold) with allied accessions based on 16S rRNA gene; Table S8: Nucleotide sequence similarity (%) for the tested strains of *Dendronalium*-like species from dumps (in bold) with allied accessions based on 16S rRNA gene; Table S9: Nucleotide sequence similarity (%) for the tested strains of *Pseudoaliinostoc*-like species from dumps (in bold) with allied accessions based on 16S rRNA gene.
